# Di-μ-chlorido-bis­[chlorido(dimethoxy­phenyl­phosphine)palladium(II)]

**DOI:** 10.1107/S1600536810012055

**Published:** 2010-04-10

**Authors:** Alexandra M. Z. Slawin, Paul G. Waddell, J. Derek Woollins

**Affiliations:** a Department of Chemistry, University of St Andrews, St Andrews KY16 9ST, Scotland

## Abstract

The title compound, [Pd_2_Cl_4_(C_8_H_11_O_2_P)_2_], is binuclear and disposed about a crystallographic centre of symmetry with a Pd⋯Pd distance of 3.4662 (17) Å. It has a similar geometry to that observed in the triphenyl­phosphite and triphenyl­phosphine analogues. The Pd—P bond length is *ca* 0.04 Å shorter than those in mononuclear PdCl_2_(P(OMe)_2_Ph)_2_, possibly due to the lower *trans*-influence of the bridging Cl^−^ compared to a single-bonded Cl^−^ atom.

## Related literature

For binuclear analogues, see: Grigsby & Nicholson (1992 ▶); Sui-Seng *et al.* (2003 ▶). For the related mononuclear palladium compound, see: Slawin *et al.* (2010 ▶).
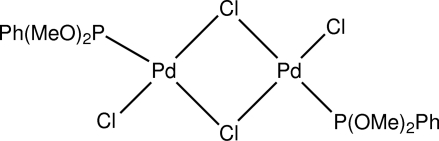


## Experimental

### 

#### Crystal data

[Pd_2_Cl_4_(C_8_H_11_O_2_P)_2_]
*M* *_r_* = 694.91
Triclinic, 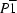

*a* = 7.078 (3) Å
*b* = 8.938 (3) Å
*c* = 9.838 (5) Å
α = 87.54 (3)°
β = 89.55 (3)°
γ = 69.46 (2)°
*V* = 582.3 (4) Å^3^
*Z* = 1
Mo *K*α radiation
μ = 2.16 mm^−1^
*T* = 125 K
0.21 × 0.12 × 0.09 mm

#### Data collection

Rigaku Mercury70 CCD diffractometer
Absorption correction: multi-scan (*ABSCOR*; Higashi, 1995 ▶) *T*_min_ = 0.591, *T*_max_ = 0.823
6130 measured reflections
2087 independent reflections
1993 reflections with *I* > 2σ(*I*)
*R*_int_ = 0.042

#### Refinement

*R*[*F* ^2^ > 2σ(*F* ^2^)] = 0.037
*wR*(*F* ^2^) = 0.075
*S* = 1.10
2087 reflections
127 parameters
H-atom parameters constrained
Δρ_max_ = 0.58 e Å^−3^
Δρ_min_ = −0.67 e Å^−3^

### 

Data collection: *SCXMini* (Rigaku, 2006 ▶); cell refinement: *SCXMini*; data reduction: *SCXMini*; program(s) used to solve structure: *SHELXS97* (Sheldrick, 2008 ▶); program(s) used to refine structure: *SHELXL97* (Sheldrick, 2008 ▶); molecular graphics: *CrystalStructure* (Rigaku, 2009 ▶); software used to prepare material for publication: *CrystalStructure*.

## Supplementary Material

Crystal structure: contains datablocks global, I. DOI: 10.1107/S1600536810012055/br2142sup1.cif

Structure factors: contains datablocks I. DOI: 10.1107/S1600536810012055/br2142Isup2.hkl

Additional supplementary materials:  crystallographic information; 3D view; checkCIF report

## Figures and Tables

**Table:** 

Pd1—P1	2.1940 (14)
Pd1—Cl2	2.2820 (15)
Pd1—Cl1^i^	2.3163 (15)
Pd1—Cl1	2.4170 (14)

**Table:** 

P1—Pd1—Cl2	86.39 (5)
P1—Pd1—Cl1^i^	95.34 (5)
Cl2—Pd1—Cl1^i^	176.98 (4)
P1—Pd1—Cl1	178.39 (4)
Cl2—Pd1—Cl1	92.45 (5)
Cl1^i^—Pd1—Cl1	85.86 (5)
Pd1^i^—Cl1—Pd1	94.14 (5)

Symmetry code: (i) 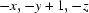.

**Fig. 1.**
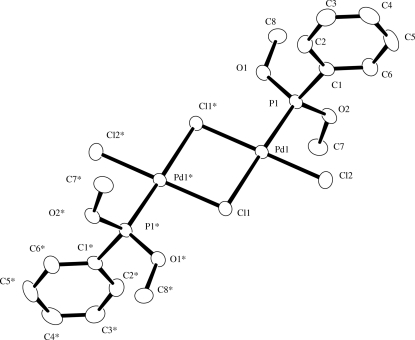
The structure of the title compound with displacement ellipsoids drawn at the 50% probability level, hydrogen atoms omitted for clarity.

## supplementary crystallographic information

### Comment

In the structure of the title compound the palladium atoms are in distorted square planar environments. The Pd-Cl bondlengths  vary with the shortest being the terminal Pd-Cl, the  longest being the bridging Pd-Cl* trans* to P and the intermediate length being for bridging Pd-Cl *trans* to Cl. This pattern is also observed in the known analogues: the triphenylphosphine analogue (Sei-Sung *et al.*, 2003) has Pd-P 2.2278 (6) Å, Pd-Cl(terminal)  2.2722 (7) Å, Pd-Cl (bridging *trans *to P) 2.4128 (6) Å and Pd-Cl (bridging *trans* to Cl) 2.3228 (6) Å whilst in the P(OPh)_3_ analogue (Grigsby & Nicholson, 1992) the values are  Pd-P 2.2187 (3), Pd-Cl(terminal)  2.269 (3) Pd-Cl (bridging* trans* to P) 2.413 (2) Pd-Cl (bridging *trans* to Cl) 2.309 (2) Å.  The Pd-P distance in the title compound (2.1940 (14) Å) is shorter than either of the above previously published structures.

### Experimental

1 g (2.6 mmol) of bis(benzonitrile)palladium(II) dichloride was dissolved in 25 ml of dichloromethane to which 0.84 ml (5.3 mmol) of dimethyl phenylphosphonite was added. The solution was stirred at room temperature for 30 mins before being filtered and then precipitated by slow addition of hexane to give a pale yellow solid. Crystals were grown for X-ray crystallography*v ia* slow diffusion of hexane into a solution of the product in dichloromethane.Yield: 0.321 g (0.46 mmol), 19 %.

### Refinement

All H atoms were included in calculated positions and refined as riding atoms with U_iso_(H) = 1.5 U_eq_. The highest peak in the difference map is 1.09 Å from atom Pd1

### Crystal data

**Table:**

[Pd_2_Cl_4_(C_8_H_11_O_2_P)_2_]	*Z* = 1
*M**_r_* = 694.91	*F*(000) = 340.00
Triclinic, *P*1	*D*_x_ = 1.982 Mg m^−^^3^
Hall symbol: -P 1	Mo *K*α radiation, λ = 0.71075 Å
*a* = 7.078 (3) Å	Cell parameters from 2478 reflections
*b* = 8.938 (3) Å	θ = 2.1–26.4°
*c* = 9.838 (5) Å	µ = 2.16 mm^−^^1^
α = 87.54 (3)°	*T* = 125 K
β = 89.55 (3)°	Prism, orange
γ = 69.46 (2)°	0.21 × 0.12 × 0.09 mm
*V* = 582.3 (4) Å^3^	

### Data collection

**Table:**

Rigaku Mercury70 CCD diffractometer	1993 reflections with *F*^2^ > 2σ(*F*^2^)
ω scans	*R*_int_ = 0.042
Absorption correction: multi-scan (*ABSCOR*; Higashi, 1995)	θ_max_ = 25.4°
*T*_min_ = 0.591, *T*_max_ = 0.823	*h* = −8→8
6130 measured reflections	*k* = −10→9
2087 independent reflections	*l* = −11→10

### Refinement

**Table:**

Refinement on *F*^2^	Secondary atom site location: difference Fourier map
*R*[*F*^2^ > 2σ(*F*^2^)] = 0.037	Hydrogen site location: inferred from neighbouring sites
*wR*(*F*^2^) = 0.075	H-atom parameters constrained
*S* = 1.10	*w* = 1/[σ^2^(*F*_o_^2^) + (0.0152*P*)^2^ + 1.7632*P*] where *P* = (*F*_o_^2^ + 2*F*_c_^2^)/3
2087 reflections	(Δ/σ)_max_ < 0.001
127 parameters	Δρ_max_ = 0.58 e Å^−^^3^
0 restraints	Δρ_min_ = −0.67 e Å^−^^3^
Primary atom site location: structure-invariant direct methods	

### Special details

**Table:**

Geometry. All esds (except the esd in the dihedral angle between two l.s. planes) are estimated using the full covariance matrix. The cell esds are taken into account individually in the estimation of esds in distances, angles and torsion angles; correlations between esds in cell parameters are only used when they are defined by crystal symmetry. An approximate (isotropic) treatment of cell esds is used for estimating esds involving l.s. planes.
Refinement. Refinement was performed using all reflections. The weighted *R*-factor (wR) and goodness of fit (*S*) are based on *F*^2^. *R*-factor (gt) are based on *F*. The threshold expression of *F*^2^ > 2.0 σ(*F*^2^) is used only for calculating *R*-factor (gt).

### Fractional atomic coordinates and isotropic or equivalent isotropic displacement parameters (Å^2^)

**Table:**

	*x*	*y*	*z*	*U*_iso_*/*U*_eq_	
Pd1	0.09659 (5)	0.37691 (4)	0.14271 (3)	0.01955 (12)	
Cl1	0.13513 (17)	0.60837 (13)	0.02692 (11)	0.0258 (3)	
Cl2	0.33715 (18)	0.36169 (14)	0.30113 (11)	0.0293 (3)	
P1	0.06174 (17)	0.16984 (13)	0.25330 (11)	0.0196 (2)	
O1	−0.1234 (5)	0.1371 (3)	0.1902 (3)	0.0247 (7)	
O2	0.0252 (5)	0.1858 (3)	0.4113 (3)	0.0251 (7)	
C1	0.2737 (7)	−0.0122 (5)	0.2477 (4)	0.0213 (9)	
C2	0.3205 (7)	−0.0794 (6)	0.1210 (5)	0.0278 (10)	
H2	0.2425	−0.0274	0.0432	0.033*	
C3	0.4803 (7)	−0.2215 (6)	0.1083 (5)	0.0337 (12)	
H3	0.5114	−0.2680	0.0221	0.040*	
C4	0.5941 (8)	−0.2954 (6)	0.2212 (5)	0.0340 (12)	
H4	0.7038	−0.3932	0.2126	0.041*	
C5	0.5504 (8)	−0.2288 (6)	0.3467 (5)	0.0352 (12)	
H5	0.6306	−0.2802	0.4238	0.042*	
C6	0.3898 (7)	−0.0872 (5)	0.3604 (5)	0.0275 (10)	
H6	0.3592	−0.0415	0.4469	0.033*	
C7	−0.1241 (8)	0.3299 (5)	0.4628 (5)	0.0323 (11)	
H7A	−0.0918	0.4235	0.4313	0.039*	
H7B	−0.1226	0.3227	0.5625	0.039*	
H7C	−0.2584	0.3403	0.4293	0.039*	
C8	−0.1806 (8)	0.0038 (6)	0.2437 (5)	0.0294 (11)	
H8A	−0.1043	−0.0933	0.1967	0.035*	
H8B	−0.3253	0.0287	0.2288	0.035*	
H8C	−0.1504	−0.0132	0.3413	0.035*	

### Atomic displacement parameters (Å^2^)

**Table:**

	*U*^11^	*U*^22^	*U*^33^	*U*^12^	*U*^13^	*U*^23^
Pd1	0.0218 (2)	0.01731 (19)	0.01916 (19)	−0.00664 (14)	0.00007 (14)	0.00122 (13)
Cl1	0.0318 (6)	0.0231 (6)	0.0250 (6)	−0.0135 (5)	−0.0074 (5)	0.0059 (4)
Cl2	0.0299 (6)	0.0328 (6)	0.0272 (6)	−0.0140 (5)	−0.0073 (5)	0.0044 (5)
P1	0.0213 (6)	0.0180 (6)	0.0182 (5)	−0.0055 (5)	0.0010 (4)	0.0018 (4)
O1	0.0244 (17)	0.0238 (17)	0.0275 (17)	−0.0109 (13)	−0.0029 (13)	0.0053 (13)
O2	0.0333 (19)	0.0188 (16)	0.0196 (15)	−0.0050 (14)	0.0041 (13)	0.0022 (12)
C1	0.024 (2)	0.019 (2)	0.022 (2)	−0.0081 (18)	0.0047 (18)	0.0003 (17)
C2	0.025 (3)	0.032 (3)	0.023 (2)	−0.006 (2)	0.0001 (19)	−0.0027 (19)
C3	0.026 (3)	0.034 (3)	0.040 (3)	−0.009 (2)	0.005 (2)	−0.011 (2)
C4	0.027 (3)	0.023 (3)	0.047 (3)	−0.003 (2)	0.012 (2)	−0.001 (2)
C5	0.032 (3)	0.034 (3)	0.032 (3)	−0.002 (2)	0.000 (2)	0.014 (2)
C6	0.028 (3)	0.029 (3)	0.023 (2)	−0.007 (2)	0.005 (2)	0.0020 (19)
C7	0.034 (3)	0.026 (3)	0.033 (3)	−0.005 (2)	0.010 (2)	−0.007 (2)
C8	0.033 (3)	0.030 (3)	0.032 (3)	−0.021 (2)	0.004 (2)	0.000 (2)

### Geometric parameters (Å, °)

**Table:**

Pd1—P1	2.1940 (14)	C3—C4	1.379 (7)
Pd1—Cl2	2.2820 (15)	C3—H3	0.9500
Pd1—Cl1^i^	2.3163 (15)	C4—C5	1.379 (7)
Pd1—Cl1	2.4170 (14)	C4—H4	0.9500
Cl1—Pd1^i^	2.3163 (14)	C5—C6	1.384 (7)
P1—O1	1.577 (3)	C5—H5	0.9500
P1—O2	1.578 (3)	C6—H6	0.9500
P1—C1	1.788 (4)	C7—H7A	0.9800
O1—C8	1.463 (5)	C7—H7B	0.9800
O2—C7	1.458 (5)	C7—H7C	0.9800
C1—C6	1.384 (6)	C8—H8A	0.9800
C1—C2	1.394 (6)	C8—H8B	0.9800
C2—C3	1.383 (7)	C8—H8C	0.9800
C2—H2	0.9500		
			
P1—Pd1—Cl2	86.39 (5)	C2—C3—H3	120.2
P1—Pd1—Cl1^i^	95.34 (5)	C5—C4—C3	120.6 (4)
Cl2—Pd1—Cl1^i^	176.98 (4)	C5—C4—H4	119.7
P1—Pd1—Cl1	178.39 (4)	C3—C4—H4	119.7
Cl2—Pd1—Cl1	92.45 (5)	C4—C5—C6	120.1 (5)
Cl1^i^—Pd1—Cl1	85.86 (5)	C4—C5—H5	120.0
Pd1^i^—Cl1—Pd1	94.14 (5)	C6—C5—H5	120.0
O1—P1—O2	107.39 (18)	C1—C6—C5	119.9 (4)
O1—P1—C1	107.02 (19)	C1—C6—H6	120.1
O2—P1—C1	101.72 (18)	C5—C6—H6	120.1
O1—P1—Pd1	108.39 (12)	O2—C7—H7A	109.5
O2—P1—Pd1	116.15 (12)	O2—C7—H7B	109.5
C1—P1—Pd1	115.53 (15)	H7A—C7—H7B	109.5
C8—O1—P1	120.3 (3)	O2—C7—H7C	109.5
C7—O2—P1	120.0 (3)	H7A—C7—H7C	109.5
C6—C1—C2	119.7 (4)	H7B—C7—H7C	109.5
C6—C1—P1	123.8 (3)	O1—C8—H8A	109.5
C2—C1—P1	116.5 (3)	O1—C8—H8B	109.5
C3—C2—C1	120.1 (4)	H8A—C8—H8B	109.5
C3—C2—H2	120.0	O1—C8—H8C	109.5
C1—C2—H2	120.0	H8A—C8—H8C	109.5
C4—C3—C2	119.7 (5)	H8B—C8—H8C	109.5
C4—C3—H3	120.2		
			
Cl2—Pd1—Cl1—Pd1^i^	177.49 (4)	O1—P1—C1—C6	−125.2 (4)
Cl1^i^—Pd1—Cl1—Pd1^i^	0.0	O2—P1—C1—C6	−12.7 (4)
Cl2—Pd1—P1—O1	173.14 (13)	Pd1—P1—C1—C6	114.0 (4)
Cl1^i^—Pd1—P1—O1	−9.34 (14)	O1—P1—C1—C2	54.7 (4)
Cl2—Pd1—P1—O2	52.20 (15)	O2—P1—C1—C2	167.2 (3)
Cl1^i^—Pd1—P1—O2	−130.28 (15)	Pd1—P1—C1—C2	−66.1 (4)
Cl2—Pd1—P1—C1	−66.83 (17)	C6—C1—C2—C3	1.0 (7)
Cl1^i^—Pd1—P1—C1	110.69 (17)	P1—C1—C2—C3	−178.9 (4)
O2—P1—O1—C8	−54.4 (3)	C1—C2—C3—C4	−0.7 (7)
C1—P1—O1—C8	54.1 (4)	C2—C3—C4—C5	−0.1 (8)
Pd1—P1—O1—C8	179.4 (3)	C3—C4—C5—C6	0.6 (8)
O1—P1—O2—C7	−74.4 (4)	C2—C1—C6—C5	−0.5 (7)
C1—P1—O2—C7	173.4 (3)	P1—C1—C6—C5	179.5 (4)
Pd1—P1—O2—C7	47.1 (4)	C4—C5—C6—C1	−0.3 (8)

Symmetry codes: (i) −*x*, −*y*+1, −*z*.

## References

[bb1] Grigsby, W. J. & Nicholson, B. K. (1992). *Acta Cryst.* C**48**, 362–364.

[bb2] Higashi, T. (1995). *ABSCOR* Rigaku Corporation, Tokyo, Japan.

[bb3] Rigaku (2006). *SCXmini Benchtop Crystallography System Software* Rigaku Americas Corporation, The Woodlands, Texas, USA.

[bb4] Rigaku (2009). *Crystal Structure* Rigaku/MSC, The Woodlands, Texas, USA, and Rigaku Corporation, Tokyo, Japan.

[bb5] Sheldrick, G. M. (2008). *Acta Cryst.* A**64**, 112–122. 10.1107/S0108767307043930 18156677

[bb6] Slawin, A. M. Z., Waddell, P. G. & Woollins, J. D. (2010). *Acta Cryst.* E**66**, m321. 10.1107/S1600536810006471 PMC2983618 21580259

[bb7] Sui-Seng, C., Bélanger-Gariépy, F. & Zargarian, D. (2003). *Acta Cryst.* E**59**, m618–m619.

